# Scrotal Abscess Induced by Spontaneous Bacterial Peritonitis Involving Enterococcus cecorum

**DOI:** 10.7759/cureus.89381

**Published:** 2025-08-04

**Authors:** Shota Fukami, Masanori Fukushima, Nozomi Ohki, Satoshi Miuma, Hisamitsu Miyaaki

**Affiliations:** 1 Department of Gastroenterology and Hepatology, Nagasaki University Graduate School of Biomedical Sciences, Nagasaki, JPN; 2 Department of Radiological Sciences, Nagasaki University Graduate School of Biomedical Sciences, Nagasaki, JPN

**Keywords:** antibiotics-resistant bacteria, ascites, enterococcus cecorum, scrotal abscess, spontaneous bacterial peritonitis

## Abstract

A 60-year-old man with idiopathic portal hypertension and ascites presented with fever, abdominal pain, and right scrotal swelling. He was diagnosed with spontaneous bacterial peritonitis (SBP) and a communicating right hydrocele, and antibiotic treatment was initiated. Despite treatment, his fever and elevated inflammatory markers persisted, accompanied by progressive genital pain. On day 21, he was diagnosed with a scrotal abscess. Owing to a poor response to antibiotics, and because scrotal ultrasound revealed a multiloculated abscess without any drainable cavity, an orchiectomy was performed on day 108. *Enterococcus cecorum*, identical to that identified in ascitic fluid, was isolated from the surgical specimen.

Communicating hydroceles associated with ascites have been reported; however, no previous reports have described scrotal abscesses resulting from SBP. In this case, antibiotic treatment was ineffective, necessitating surgical excision. This case highlights the importance of careful monitoring of patients with SBP and hydrocele, as scrotal abscesses may develop.

## Introduction

Scrotal hydrocele is a relatively rare complication of ascites, and many cases reportedly improve with ascites treatment or the correction of hypoalbuminemia [1]. In adults, communicating scrotal hydrocele often results from a patent processus vaginalis [2]. Scrotal abscesses can develop when the ascitic fluid is infected with generalized peritonitis, such as perforated appendicitis, and accumulate in the scrotum via the inguinal canal [3].

Here, we report a case of scrotal abscess resulting from *Enterococcus cecorum* (*E. cecorum*) infection following spontaneous bacterial peritonitis (SBP). The abscess was challenging to drain, was non-responsive to antibiotic treatment, and necessitated the surgical removal of the scrotal contents. Reports of scrotal abscesses resulting from SBP and those of *E. cecorum *as the causative organism of SBP are extremely rare. Herein, we describe the clinical course and treatment in this case.

## Case presentation

A 60-year-old man presented to the emergency department with fever, abdominal pain, and right scrotal enlargement. Ten years earlier, the patient was found to have esophageal varices, which prompted further evaluation for portal hypertension. Despite a comprehensive workup, including imaging and laboratory tests, no underlying liver disease, such as viral hepatitis or autoimmune hepatitis, was identified. Eight years ago, he underwent splenectomy due to concurrent idiopathic thrombocytopenic purpura (ITP). Histological examination of the liver at that time showed preserved architecture without bridging fibrosis or cirrhosis. Based on the presence of portal hypertension in the absence of cirrhosis or other identifiable causes, a diagnosis of idiopathic portal hypertension (IPH) was made. Since then, he has been regularly followed up for ascites control and surveillance of esophageal varices.

**Figure 1:**
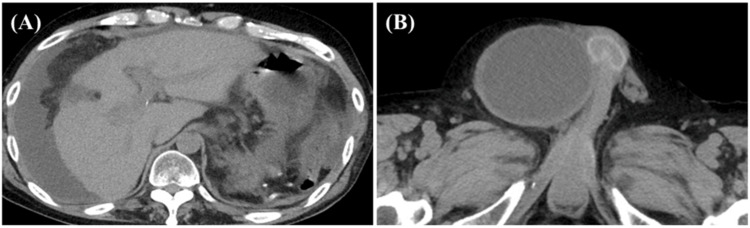
Computed tomography (CT) images at the time of admission (A) The liver exhibited an irregular surface with a large volume of ascitic fluid, along with increased density of intraperitoneal fat strands. CT revealed no evidence of intestinal perforation or other localized intra-abdominal infection. (B) A communicating right scrotal hydrocele was suspected.

The results of blood tests taken at the time of initial consultation showed a white blood cell (WBC) count of 9,600/µL (normal range: 3300-8600/µL), neutrophils 84.0%, lymphocytes 7.0%, and a C-reactive protein (CRP) level of 20.7 mg/dL (normal range: 0.00-0.14 mg/dL), indicating a high inflammatory response. Additional findings were as follows: total bilirubin, 1.7 mg/dL (normal range: 0.4-1.5 mg/dL); albumin, 2.4 g/dL (normal range: 4.1-5.1 g/dL); prothrombin time, 69% (PT-INR, 1.24); and a Child-Pugh score of 10 (Class C). Paracentesis revealed turbid ascitic fluid with a polymorphonuclear leukocyte count of 2,187/mm³. The serum ascites albumin gradient was 1.5 (>1.1), consistent with portal hypertension-related ascites. The ascitic fluid lactate dehydrogenase (LDH)/serum LDH ratio was 1.09 (>0.6), suggestive of exudative ascites. The patient was diagnosed with SBP and a right hydrocele. CT revealed no evidence of intestinal perforation or other localized intra-abdominal infection (Figure 1). Intravenous (IV) cefmetazole therapy was initiated. Ascitic WBCs normalized, and the fluid volume decreased following drainage; however, the right scrotal enlargement persisted.

**Figure 2:**
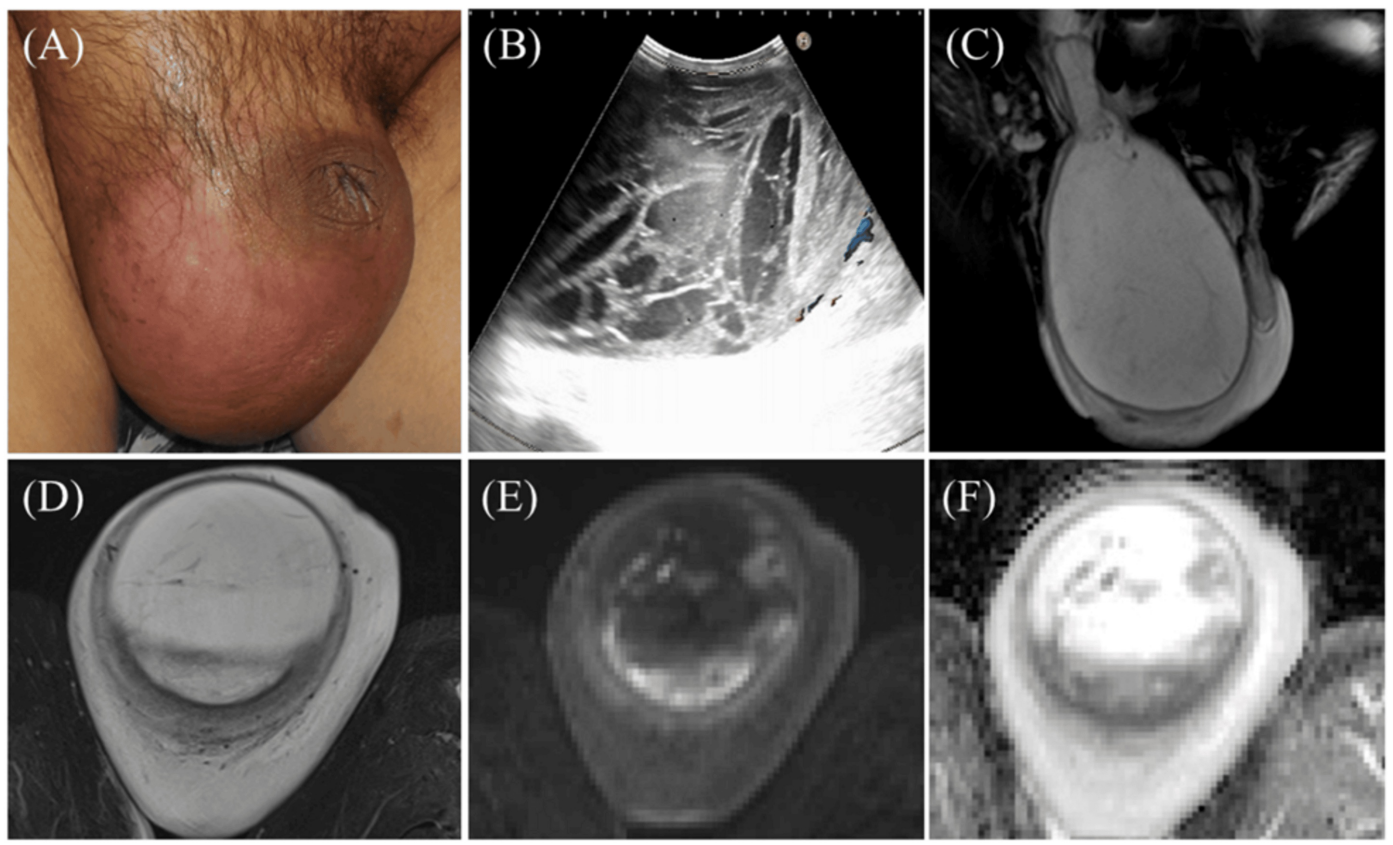
Imaging of the scrotum (A) The scrotum, erythematous predominantly on the right side, is markedly swollen to approximately the size of a clenched fist. (B) Scrotal ultrasound revealing multiple septa within the scrotum, with no well-defined abscess cavity suitable for drainage. (C) Coronal fat-suppressed T2-weighted image showing a large cystic lesion (size, 22 cm) extending from the right inguinal canal into the scrotum. (D) Axial fat-suppressed T2-weighted image showing a bilocular lesion, with a thickened cyst wall and internal low-signal linear structures suggestive of septations. Surrounding the cyst wall, edematous changes in the adjacent soft tissue are also observed. (E) Diffusion-weighted imaging (b = 1000 s/mm^2^) demonstrating increased signal intensity predominantly along the cyst wall, appearing as punctate and sheet-like areas. (F) The corresponding apparent diffusion coefficient map shows low signal intensity in these regions, indicating restricted diffusion consistent with an abscess.

Owing to the prolonged fever and elevated CRP levels, his treatment was switched to IV piperacillin/tazobactam on day 10. During treatment, his genital pain worsened, and the scrotum became firm and erythematous (Figure 2A). On day 21, a right scrotal infection was diagnosed, and he was then switched to IV ceftriaxone + vancomycin. Despite repeated blood, ascitic fluid, and scrotal content cultures for general and acid-fast bacteria, all results remained negative.

Ultrasound examination findings revealed multiple septa within the scrotum but no distinct abscess cavity suitable for drainage; therefore, drain tube placement was abandoned (Figure 2B). On day 33, IV minocycline (MINO) was added to the target-resistant and intracellular bacteria. On day 42, as the symptoms and inflammatory markers improved, the treatment was switched to oral MINO, and the patient was discharged. However, on day 98, the patient was readmitted with recurrent abdominal and scrotal pain and increased white WBCs in the ascitic fluid. Worsening SBP and scrotal infection were diagnosed, and IV ceftriaxone was restarted. *Enterococcus* *cecorum* was detected in the ascitic fluid. Given its antibiotic sensitivity, ampicillin was added (Table 1).

**Table 1:** Sensitivity of Enterococcus cecorum to antibiotic treatment MIC, minimum inhibitory concentration; I, intermediate; S, susceptible; R, resistant.

Antibiotic	MIC	Susceptibility
Oxacillin	>2	R
Ampicillin	≤1	S
Ampicillin/Sulbactam	≤2/1	S
Amoxicillin/Clavulanic Acid	≤2/1	S
Cefazolin	≤2	R
Cefotiam	4	R
Cefbuperazone	8	R
Cefmetazole	8	R
Cefditoren Pivoxil	>2	R
Latamoxef	>32	R
Imipenem	≤0.5	S
Meropenem	≤0.5	S
Gentamicin	8	R
Amikacin	16	R
Arbekacin	>8	R
Erythromycin	>4	R
Clindamycin	>2	R
Minocycline	8	I
Chloramphenicol	8	S
Vancomycin	≤0.5	S
Teicoplanin	≤0.5	S
Sulfamethoxazole/Trimethoprim	>4/76	R
Moxifloxacin	>1	R
Linezolid	2	S
Daptomycin	≤0.5	S
Gentamicin 500	≤500	S

Despite the ascitic WBCs having rapidly normalized, scrotal enlargement, fever, and inflammatory markers persisted. Testicular magnetic resonance imaging (Figure 2C-2F) revealed multiple areas of restricted diffusion, suggesting abscess formation. As the scrotal infection remained uncontrolled, and antibiotic therapy was deemed ineffective, excision of the scrotal contents was performed on day 108. *Enterococcus* *cecorum*, identical to that in the ascitic fluid, was cultured from the surgical specimen.

**Figure 3:**
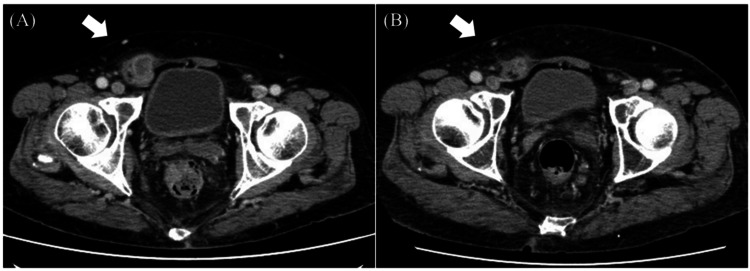
Computed tomography (CT) images after excision of the scrotal contents (A) CT scans showed wall thickening near the inguinal canal with surrounding adipose tissue inflammation (arrow). (B) Following the administration of oral ampicillin and minocycline, a reduction in inflammation was observed, as evidenced by the CT findings (arrow).

**Figure 4:**
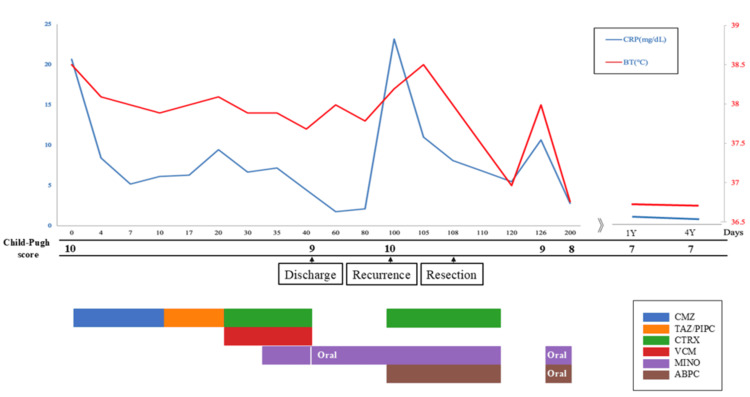
Treatment progress chart The graph illustrates the temporal progression of body temperature, C-reactive protein levels, and associated treatment details. BT, body temperature; ABPC, aminobenzylpenicillin (ampicillin); CMZ, cefmetazole; CTRX, ceftriaxone; MINO, minocycline; TAZ/PIPC, tazobactam/piperacillin; VCM, vancomycin.

On day 126, the patient developed fever and elevated CRP levels again. CT scans showed wall thickening near the inguinal canal with surrounding adipose tissue inflammation, suggestive of a recurrent infection (Figure 3). The patient was treated with oral ampicillin and oral MINO for three months. No recurrence occurred during the four-year follow-up period (Figure 4).

## Discussion

Here, we report a rare case of a scrotal abscess triggered by SBP. *Enterococcus cecorum*, which was the causative pathogen, is primarily an enterobacterium found in livestock, with few reports of human infections. The strain was resistant to cephem, necessitating surgical intervention.

Scrotal abscesses that develop from SBP are exceedingly rare, with only one similar case having been reported [4]. SBP is a common infection in patients with cirrhosis and ascites and is associated with a poor prognosis in 10-30% of cases [5]. Scrotal abscesses can result from epididymo-orchitis, neglected testicular torsion, spread of intra-abdominal abscesses via an open peritoneal sheath, or hematogenous or idiopathic infections [6]. Cases of scrotal abscesses owing to intraperitoneal abscess spillover from a perforated appendicitis have also been reported [7]. The processus vaginalis remains patent in 70-80% of newborns, with 90% closing by age two years and approximately 10% persisting into adulthood [8,9]. This anatomical feature suggests that purulent ascites from SBP can accumulate in the scrotum via the inguinal canal.

In this instance, the initial CT findings indicated the presence of significant ascites and scrotal edema. During treatment for SBP, the patient experienced prolonged fever and gradually reported scrotal pain. This clinical progression suggested the development of a scrotal abscess as a consequence of SBP. The diagnosis was confirmed following the detection of *E. cecorum* in both ascitic fluid and scrotal abscess cultures.

*Enterococcus cecorum* was first identified as part of the poultry intestinal flora in 1983 [10]. *Enterococcus cecorum* is generally considered an opportunistic pathogen that primarily causes infections in immunocompromised individuals or patients with significant comorbidities, such as malignancies and liver cirrhosis, or organ failure [11]. Human infections remain rare, with only eight reported cases primarily involving intra-abdominal infections such as SBP and peritoneal dialysis-related peritonitis [11-17]. In our case, the SBP was similar to that in other cases. The pathway of infection remained unidentified as there was no pet ownership or history of raw meat consumption (Table 2). Research on poultry-derived strains of *E. cecorum* has shown that most of these strains can withstand heating at 60°C for 60 min, but are eliminated at temperatures between 70 and 75°C. This suggests that consuming undercooked meat may also present a risk [18]. This case concerns a patient who underwent a splenectomy due to ITP and IPH. Although *E. cecorum* lacks a polysaccharide capsule, the immunological effects of asplenia may have contributed to the infection.

**Table 2:** Reported infections caused by Enterococcus cecorum, identified using PubMed (1983-2024) SBP, spontaneous bacterial peritonitis; CAPD, continuous ambulatory peritoneal dialysis; HBV, hepatitis B virus.

Case	Age (years)	Sex	Disease	Medical history	History of proton pump inhibitor use	History of pet ownership	Infection source	Cultures\specimen	Treatment	Treatment duration	Outcome
Our case	61	M	Scrotal abscess	Idiopathic portal hypertension	Yes	No	Unknown	Ascites	Cefmetazole etc.	108 days	Improved
			SBP recurrence					Scrotal contents	Scrotal content excision		
1 (Ahmed et al. [11])	58	M	Aortic valve endocarditis	None	No	No	Oral infection suspected	Blood	Amoxicillin	45 days	Improved
								Aortic valve	Gentamicin		
									Aortic valve replacement		
2 (Woo et al. [13])	44	M	Pyothorax	Decompensated liver cirrhosis	Unknown	Unknown	Unknown	Pleural fluid	Cefotaxime	10 days	Improved
			SBP recurrence	(Wilson's disease）					Abscess drainage		
3 (Delaunay et al. [14])	56	M	Incisional hernia plate infection	Crohn's disease	Unknown	Unknown	Unknown	Incisional hernia plate	Amoxicillin	40 days	Improved
									Surgery		
4 (Delaunay et al. [14])	39	F	Asymptomatic bacteriuria	Post-kidney transplantation	Unknown	Unknown	Unknown	Urine	Observation	Unknown	Unchanged
5 (Pang et al. [15])	72	M	Suspected SBP	Decompensated liver cirrhosis	Yes	Unknown	Unknown	Blood	Ceftriaxone	10 days	Improved
				(alcoholic)							
6 (De Baere​​​​​​​ et al. [16])	44	M	CAPD-related peritonitis	Decompensated liver cirrhosis	Unknown	Unknown	Unknown	CAPD effluent	Cefazolin	10 days	Improved
				(alcoholic)					Gentamicin		
				Hepatorenal syndrome							
7 (Hsueh​​​​​​​ et al. [17])	60	M	Septicemia	Decompensated liver cirrhosis	Unknown	Unknown	Unknown	Blood	Cefoxitin	53 days	Deceased
			SBP recurrence	(HBV-positive)				Ascites			
				Hepatocellular carcinoma							
8 (Greub​​​​​​​ et al. [12])	44	F	Septicemia	Morbid obesity (postoperative)	Unknown	Yes	Percutaneous infection suspected	Blood	Imipenem	21 days	Improved
				Vitamin deficiency							
				(with skin lesions)							

Initial SBP treatment with cefmetazole failed because *E. cecorum* was resistant to cephem antibiotics. This may have led to the formation of scrotal abscesses. Once developed, scrotal abscesses necessitate drainage or surgical intervention owing to inadequate antimicrobial agent penetration, even when appropriate antibiotics are selected. It has been reported that 27% (4/15) of scrotal abscesses require surgical intervention [19]. The blood-testis barrier often limits drug penetration, leading to antibiotic resistance [20]. Drainage or surgical excision should be considered when fever persists despite antibiotic therapy.

In this case, scrotal drainage was discontinued because of the presence of multiple septal walls and the challenges associated with accurately identifying the position of the testes during ultrasound examination of the scrotum. Early drain placement may have prevented the need for surgical intervention. Given the potential for further accumulation of ascites owing to an underlying disease, it would have been necessary to ligate the open peritoneal sheath process to prevent recurrence. Additional case series are required to determine the frequency of occurrence, preventive strategies, and long-term prognosis.

## Conclusions

A case of scrotal abscess secondary to SBP was observed, with *E. cecorum *resistant to cephem antibiotics identified as the causative organism. While rare, *E. cecorum* has been reported as a causative pathogen of peritonitis. In patients who do not respond to empirical therapy, repeated microbiological identification is essential. Attention should also be given to the potential progression from SBP to scrotal hydroceles and ultimately to scrotal abscesses.
